# The NLRP3 inflammasome in physiological and dysfunctional host response in human sepsis and critical illness: a narrative review

**DOI:** 10.1186/s13054-026-06315-z

**Published:** 2026-09-19

**Authors:** Caroline Neumann, Florian Hickl, Julia Kemnitzer, Margit Leitner, Evangelos J. Giamarellos-Bourboulis, Ignacio Rubio

**Affiliations:** 1 Department of Anaesthesiology and Intensive Care Medicine, Jena University Hospital, Am Klinikum 1, 07747 Jena, Germany; 2 Center for Sepsis Control and Care, Jena University Hospital, Jena, Germany; 3 Hellenic Institute for the Study of Sepsis, Athens, Greece; 4 Department of Internal Medicine, National and Kapodistrian University of Athens Medical School, Athens, Greece

**Keywords:** NLRP3, Inflammasome, PAMPs (pathogen-associated molecular patterns), DAMPs (damage-associated molecular patterns), Interleukin-1β (IL-1β), Interleukin-18 (IL-18), Sepsis, Hyperinflammation, Immune paralysis, Therapeutic targets

## Abstract

**Background:**

The NLRP3 inflammasome is a cytosolic multiprotein complex that serves as a key regulator of innate immunity by reacting to pathogen- and damage-associated signals and orchestrating downstream inflammatory responses. Inflammasome activation induces caspase-1-dependent maturation of interleukin (IL)-1β, IL-18 and gasdermin D, which results in pyroptotic cell death.

**Main text:**

While tightly controlled inflammasome activation is essential for effective host defence and pathogen clearance, accumulating evidence implicates its dysregulation as a central driver of host response imbalance in critical illness. In conditions such as sepsis and acute respiratory distress syndrome, excessive activation contributes to hyperinflammation, endothelial dysfunction, immunothrombosis, and multi-organ failure. Conversely, insufficient or exhausted inflammasome responses may impair microbial clearance and predispose to secondary infections. These observations underscore the context-dependent and temporally dynamic role of inflammasome signalling as being either protective or pathogenic. Emerging therapeutic strategies aim to restore immune homeostasis through targeted modulation of inflammasome pathways, including IL-1 blockade (e.g., anakinra) and inhibition of upstream signalling components. However, clinical translation remains challenging, requiring improved patient stratification, biomarker-guided approaches, and a deeper understanding of disease heterogeneity.

**Conclusions:**

Inflammasome signalling plays a highly dynamic role in critical illness, contributing to effective host defence, if functional, or to pathological inflammation, if dysregulated. Although targeted modulation of inflammasome pathways represents a promising therapeutic strategy, further research is needed to refine patient selection, identify reliable biomarkers, and better characterise disease heterogeneity to enable successful clinical translation.

**Graphical Abstract:**

**Figure:**
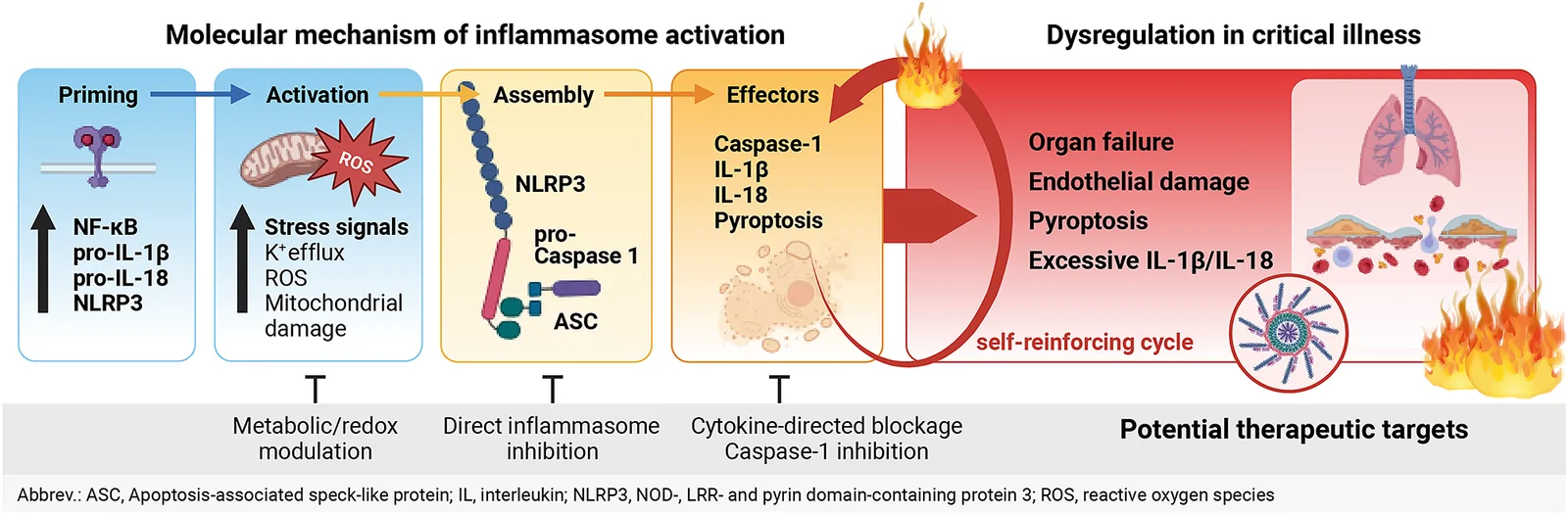

## Background

Critical illness, including conditions such as sepsis, acute respiratory distress syndrome (ARDS), and multiple organ dysfunction syndrome (MODS), remains a major cause of morbidity and mortality worldwide despite advances in supportive care [1]. A unifying feature across these syndromes is not only the presence of infection or tissue injury but the host’s dysregulated response to these insults. This concept, formally incorporated into the definition of sepsis [2], reflects a paradigm shift away from viewing sepsis as a purely excessive inflammatory response toward a more complex model of host defence failure, including immune dysregulation.

Rather than a linear transition from an initial hyperinflammatory phase to subsequent immunosuppression, critically ill patients frequently exhibit simultaneous pro- and anti-inflammatory processes [3, 4]. This host response imbalance is characterised by profound heterogeneity in immune activation across time, compartments, and individuals, contributing to variable clinical trajectories and outcomes. Understanding the molecular mechanisms that govern these processes is essential for the development of effective, targeted therapies.

The inflammasome, particularly NLRP3, is a key innate immune complex that detects pathogenic and damage signals, triggering inflammatory cytokine release and pyroptosis in response to various stimuli relevant to critical illness [5, 6].

Although several other sensors can assemble and activate the inflammasome, NLRP3 has emerged as the most extensively studied and clinically relevant representative of this group because it integrates a broad range of danger signals and currently has the strongest mechanistic and translational evidence in sepsis and critical illness [5, 7]. Consequently, this review primarily focuses on NLRP3.

In physiological conditions, inflammasome activation contributes to effective host defence by promoting pathogen clearance and coordinating early inflammatory responses [6]. However, when dysregulated in magnitude, duration, or spatial distribution, inflammasome signalling can drive maladaptive inflammation, where excessive activation drives endothelial dysfunction, immunothrombosis, and tissue injury [8, 9]. Conversely, insufficient or exhausted inflammasome activity may impair microbial clearance and predispose patients to secondary infections [7]. These observations position the inflammasome as a key determinant of an effective host response.

Recent advances in the understanding of inflammasome biology have generated considerable interest in its therapeutic targeting [10–12]. Strategies aimed at modulating inflammasome activity hold promise for restoring immune homeostasis [13], including IL-1 blockade [12, 14, 15] and direct inhibition of upstream signalling pathways. However, clinical translation remains challenging, reflecting the complexity of immune dysregulation in critical illness, including issues related to patient heterogeneity, timing of intervention, and the lack of robust biomarkers to guide therapy [16].

In this narrative review, we aim to (i) delineate the role of inflammasome activation in physiological host defence and in the dysregulated immune response characteristics of critical illness, (ii) integrate current mechanistic and clinical evidence across conditions such as sepsis and ARDS, (iii) critically evaluate emerging therapeutic strategies targeting inflammasome pathways, and (iv) identify key challenges and future directions for precision immunomodulation in critically ill patients.

To identify the relevant literature, we conducted a targeted search of PubMed, Embase, and Web of Science for publications addressing the NLRP3 inflammasome, innate immune responses, and their relevance to sepsis and critical illness, including associated organ dysfunction, biomarkers, and therapeutic strategies. Original research, clinical trials, observational and translational studies, and relevant reviews were considered. A particular emphasis was placed on recent high-quality clinical investigations, landmark mechanistic studies, and studies directly relevant to inflammasome signalling in critical illness. Furthermore, reference lists of selected publications were screened to identify relevant studies not captured by the database search.

## Methods

This narrative review summarizes and draws conclusions from the current evidence on the role of the NLRP3 inflammasome in physiological host responses and its dysregulation in sepsis and critical illness. Literature search was performed using PubMed, Embase, and Web of Science to identify relevant publications. The search included articles available online up to March 2026 and used combinations of the following keywords: *acute kidney injury*, *acute lung injury*, *ARDS*, *biomarker*, *brain injury*, *caspase-1*, *critical care*, *critical illness*, *cytokines*, *DAMPs*, *encephalopathy*, *gasdermin D*, *host response*, *Il-1β*, *Il-18*, *immune dysregulation*, *immunotherapy*, *inflammation*, *inflammasome*, *innate immunity*, *intensive care unit*, *ischaemia reperfusion injury*, *MODS*, *NLRP3*, *organ dysfunction*, *outcome*, *PAMPs*, *pyroptosis*, *sepsis*, *surgery*, *therapy*, and *trauma*.

Original research articles, clinical trials, observational studies, translational studies, and relevant review articles published in English were considered. Priority was given to recent studies, high-quality clinical investigations, landmark mechanistic studies, and publications with direct relevance to the role of inflammasome signalling in critical illness. References of selected articles were also screened to identify additional relevant literature.

Articles focusing exclusively on (chronic) inflammatory diseases without relevance to critical illness, conference abstracts without sufficient methodological detail, and non-English publications were generally excluded. Animal studies were not included unless they provided essential mechanistic insights directly relevant to inflammasome biology in critical illness. Study selection was based on the authors’ assessment of scientific quality, relevance, and contribution to the aims of this narrative review.

### The inflammasome in the physiological host response: basic concepts and molecular mechanisms

**Fig. 1:**
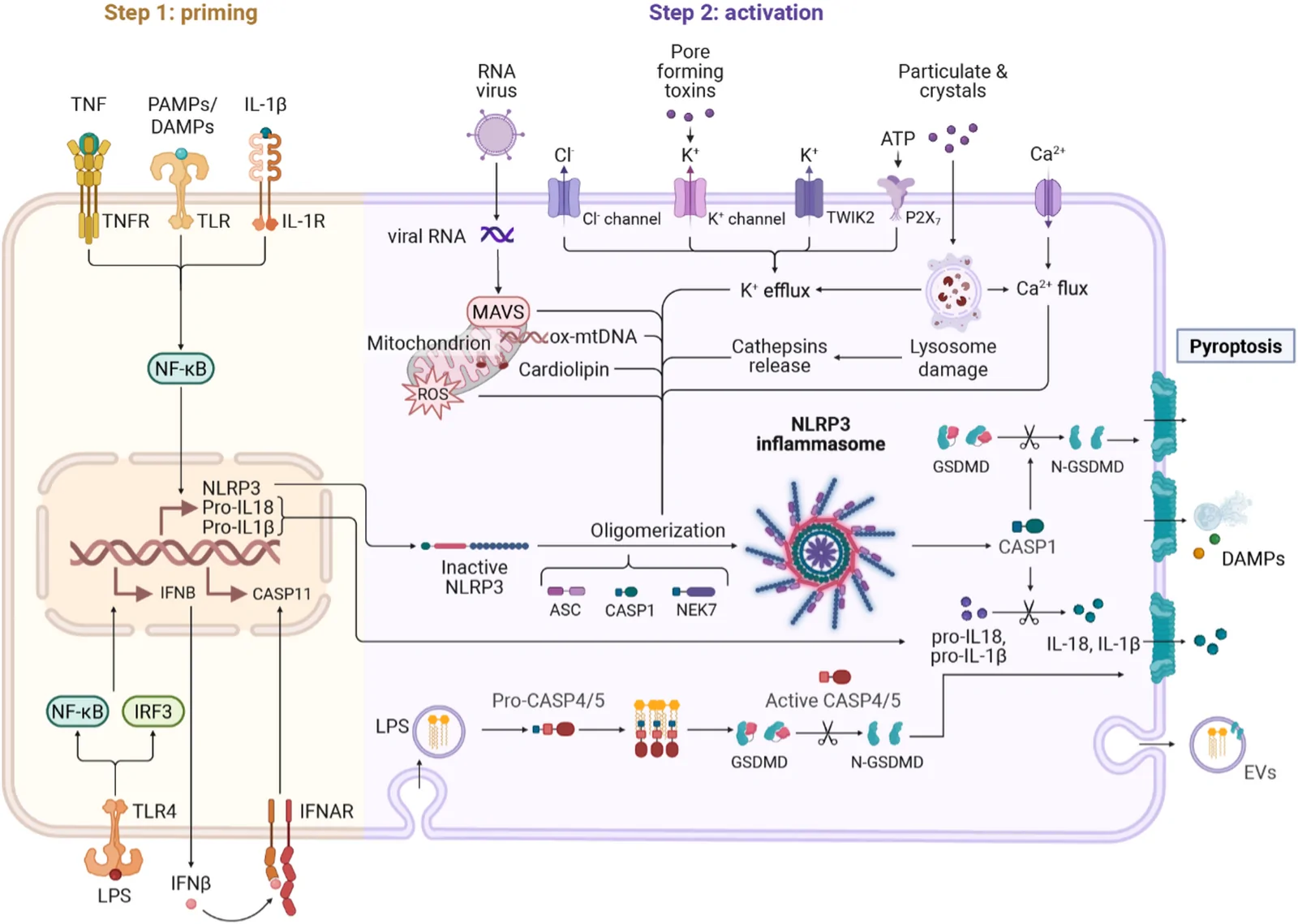
Two-step activation of the NLRP3 inflammasome in innate immune cells. In the priming step, receptor signalling activates NF-κB, leading to production of NLRP3 and precursors of pro-inflammatory cytokines. In the activation step, cellular stress signals, such as ion flux, ATP, ROS, mitochondrial damage or uric acid crystals, trigger assembly of the inflammasome complex. This results in caspase-1 activation, cytokine maturation (IL-1β, IL-18), and the induction of pyroptosis via GSDMD-mediated pore formation. GSDMD pores allow the release of IL-1β, IL-18 and numerous DAMPs, many of which are likely to be yet unknown. Abbreviations: ASC: apoptosis-associated speck-like protein containing a caspase recruitment domain; ATP: adenosine triphosphate; CASP: caspase; DAMPs: damage-associated molecular patterns; EVs: extracellular vesicles; GSDMD: gasdermin D; IFNAR: interferon-α/β receptor; IFNB: interferon beta; IL: interleukin; IL1R: interleukin-1 receptor; LPS: lipopolysaccharide; MAVS: mitochondrial antiviral-signalling protein; NEK7: NIMA-related kinase 7; NLRP3: NOD-, LRR- and pyrin domain-containing protein 3; oxmtDNA: oxidized mitochondrial DNA; P2 × 7: purinergic receptor P2X ligand-gated ion channel 7; TWIK2: tandem of P domains in a weak inwardly rectifying K⁺ channel 2; TNF: tumor necrosis factor; PAMPs: pathogen-associated molecular patterns; TLR: Toll-like receptor; TNFR: tumor necrosis factor receptor; ROS: reactive oxygen species. Created with BioRender.com

Inflammasomes are cytosolic multiprotein complexes that serve as central effectors of the innate immune system, enabling rapid detection of PAMPs (pathogen-associated molecular patterns) and DAMPs (damage-associated molecular patterns) to elicit an inflammatory reaction with the aim of preserving tissue integrity. Canonical inflammasomes, including members of the NLR family such as NLRP3 (NOD-, LRR- and pyrin domain-containing protein 3; cryopyrin) or NLRC4 (NLR family CARD domain-containing protein 4) and AIM2 (absent in melanoma 2), are typically activated downstream of extracellular danger signals sensed by pattern recognition receptors (PRR) such as Toll-like receptors (TLR). Non-canonical inflammasomes, represented by the human Caspase-4/5 cassette, primarily detect intracellular pathogens or PAMPs. Inflammasome activation follows a tightly controlled two-step process [6, 17] (Fig. 1). Priming signals, provided by DAMPs/PAMPs and transmitted via NF-κB (Nuclear Factor kappa-light-chain-enhancer of activated B cells), induce the transcription of inflammasome components (e.g., NLRP3, pro-caspase-1) and precursors of pro-inflammatory cytokines, such as pro-IL-1β and pro-IL-18. A second activation signal, triggered by cellular stressors that indicate damage, infection, or loss of homeostasis or signals of tissue breakdown, including potassium efflux, mitochondrial dysfunction, cytosolic nucleic acids or crystalline particles, promotes the assembly of the inflammasome complex around the adaptor ASC (Apoptosis-associated speck-like protein), forming the so-called ASC speck. This assembly facilitates autocatalytic activation of caspase-1 (and related caspases), resulting in cleavage of pro-IL-1β and pro-IL-18 into their bioactive forms and concomitant activation of gasdermin D (GSDMD), a pore-forming protein that incorporates into the plasma membrane, causing cytokine release and pyroptotic cell death [18, 19]. Through this coordinated response, inflammasomes eliminate intracellular replication niches and amplify local inflammation, thereby promoting recruitment and activation of neutrophils and monocytes.

Beyond their immediate antimicrobial effects, inflammasomes play a critical role in bridging innate and adaptive immunity. IL-1β and IL-18 enhance dendritic cell maturation, antigen presentation, and subsequent T cell priming, particularly promoting Th1 and Th17 differentiation [20, 21]. In addition, IL-18 stimulates natural killer and T cells to produce interferon-γ [22, 23], strengthening host defence against intracellular pathogens. In physiological settings, inflammasome activity contributes to tissue homeostasis by regulating inflammatory tone, facilitating clearance of damaged or infected cells, and supporting tissue repair. Importantly, inflammasome signalling extends beyond IL-1β and IL-18: pyroptotic cell death results in the release of additional DAMPs, such as HMGB1 (High Mobility Group Box 1), progranulin or galectin-1 [24–26], which can further amplify and propagate inflammatory responses. Inflammasome activation is not restricted to immune cells but can also occur in parenchymal tissues, and its effects may disseminate systemically via circulating immune cells or extracellular vesicles carrying PAMPs or active inflammasome components [27–29], thereby coordinating responses beyond the initial site of infection.

A defining feature of inflammasome biology is its multilayered regulation, which ensures rapid and accurate, yet self-limited and tightly controlled activation. In addition to the requirement for priming and activation signals, regulatory mechanisms, such as autophagy, ubiquitination, and spatial confinement to infected niches, prevent excessive or chronic activation. This tight control is essential, as the biological consequences of inflammasome activation are highly context-dependent and can vary according to pathogen type, tissue environment, and host factors. Experimental and clinical evidence underscores the crucial role of specific inflammasomes in different challenges to the host defence. NLRC4 is engaged in the clearance of flagellated bacteria [30], while AIM2 mediates recognition of cytosolic DNA [31, 32]. In contrast, the prototypical NLRP3 is involved in multiple scenarios of viral and bacterial infection. In line with its key role in host defence, dysregulated or excessive inflammasome activation can drive immunopathology. In humans, gain-of-function variants of inflammasome components lead to autoinflammatory syndromes, such as cryopyrin-associated periodic syndromes (CAPS) [33, 34], while aberrant NLRP3 activation has been implicated in diseases such as gout and atherosclerosis; conversely, reduced inflammasome function increases susceptibility to infection.

Collectively, the inflammasome represents a tightly regulated system that integrates pathogen sensing, inflammatory signalling, and cell death to orchestrate host defence. While controlled activation is essential for effective immunity and maintenance of tissue homeostasis, dysregulation can result in excessive systemic inflammation, contributing to the pathogenesis of critical conditions, such as sepsis.

### The inflammasome in critically ill patients: transition from protection to harm

#### Inflammasome activation in critical illness

Inflammasome activation is a key interface, where protective immunity may transition into detrimental maladaptive inflammation in critical illness [7, 35].

In critically ill patients, the inflammasome can be triggered in conditions such as trauma, burns, ischemia–reperfusion injury or major surgery [36–43]. These signals are detected by PRRs, particularly their best-known representatives, the TLRs and initiate NLRP3 inflammasome priming via the canonical pathway [5, 7, 35, 44]. After priming, the detection of intracellular stress signals, such as reactive oxygen species (ROS), potassium efflux or mitochondrial dysfunction, which are prevalent in critically ill patients, leads to the final activation of the NLRP3 inflammasome [45–49]. Furthermore, the non-canonical and alternative inflammasome pathways mediate one-step activation by TLR ligands [50–54].

While initially protective by promoting pathogen clearance and immune coordination, inflammasome activity frequently becomes dysregulated in critical illness [7, 10, 50, 55]. Hyperactivation results in excessive activation of caspase-1, which consequently leads to the excessive maturation of IL-1β and IL-18. Elevated levels of these proinflammatory cytokines are associated with increased disease severity and mortality in critically ill patients [56–60]. A key pathological feature is the disruption of endothelial integrity, associated with destruction of the glycocalyx layer. This leads to capillary leak and vasoplegia, thereby causing extravascular volume shift and hypotension as well as hypoperfusion of tissues [61–64]. In addition, released components from the destroyed glycocalyx act as DAMPs and promote further systemic inflammation [41, 65, 66]. Endothelial and glycocalyx damage create procoagulant surfaces, which, in combination with blood stasis, greatly facilitate microvascular thrombosis. Moreover, IL-1β production by the inflammasome leads to tissue factor-based activation of the coagulation system and contributes to the activation of platelets. Together, these mechanisms further increase vascular occlusion, organ hypoperfusion and injury [9, 67–71].

Caspase-1-dependent activation of the pore-forming protein gasdermin D (GSDMD), which predominantly results in pyroptosis, a type of regulated cell death (RCD) initiated by GSDMD pores, that directly damages affected tissues [18]. Pyroptotic cell lysis furthermore releases proinflammatory cytokines as well as DAMPs, thus contributing to the propagation of inflammasome activation to neighbouring cells und ultimately to increased systemic inflammation [72]. The magnitude of pyroptosis has been shown to correlate with the severity of illness and mortality in patients with sepsis [73]. In addition to pyroptosis, other forms of RCD, i.e., necroptosis, ferroptosis, panoptosis and netosis, are also closely linked to inflammasome activity and inflammation-associated tissue damage [56, 72, 74–77]. Specific combinations of RCD signatures, i.e., co-occurrence of pyroptosis and ferroptosis, have been reported to correlate significantly with higher mortality [56]. However, under specific circumstances, such as cellular hyperactivation characterized by limited GSDMD pore formation and efficient membrane repair (e.g., via ESCRT-III), GSDMD can mediate the release of pro-inflammatory mediators while preserving cell viability and without inducing overt pyroptotic cell death [78–80].

Paradoxically, excessive or dysregulated inflammasome activation can not only amplify inflammatory responses but may also contribute to subsequent immunosuppressive states. This phenomenon reflects the complex balance between inflammatory activation and compensatory mechanisms that limit excessive tissue damage. Such immune reprogramming can impair antimicrobial functions, including monocyte/macrophage responsiveness, antigen presentation, and cytokine production, thereby contributing to impaired pathogen clearance and increased susceptibility to secondary infections, even when the initial trigger of inflammasome activation was non-infectious [81, 82]. The resulting persistence or accumulation of pathogens may further aggravate organ damage [83–90]. This concept is supported by observations in patients with Gram-negative sepsis, in whom ex vivo stimulation of blood mononuclear cells demonstrated reduced IL-1β production, particularly among patients with higher disease severity, suggesting impaired inflammasome responsiveness during severe disease. Similarly, experimental endotoxemia studies have demonstrated a transient suppression of NLRP3 inflammasome activity, assessed by caspase-1 activation, following endotoxin exposure, followed by gradual restoration over time [91]. These findings indicate that excessive inflammatory stimulation can induce a state of temporary inflammasome hyporesponsiveness, representing a potential mechanism contributing to impaired host defence during critical illness.

### Organ-specific impact of inflammasome activation

**Fig. 2:**
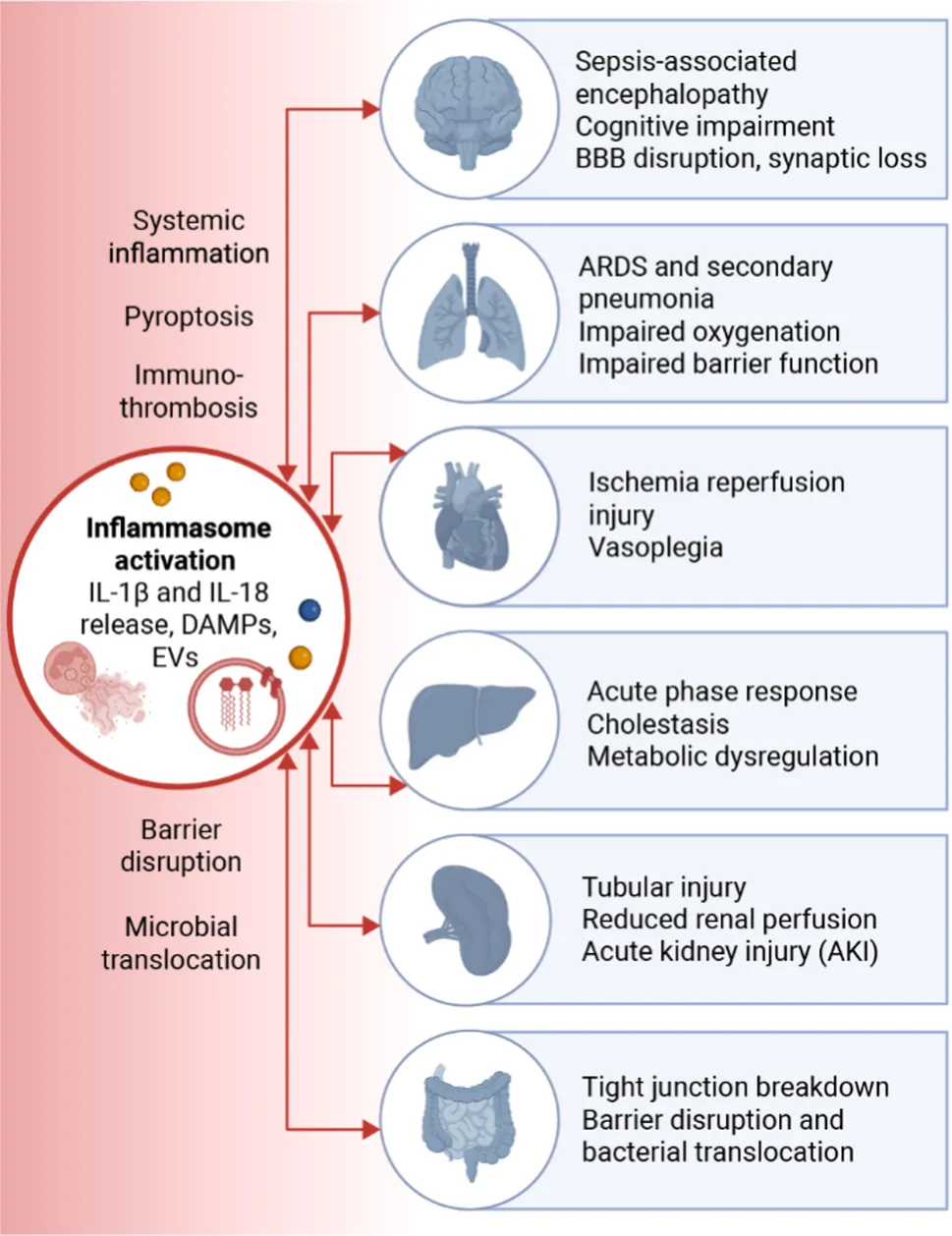
NLRP3-driven tissue injury and organ dysfunction in critical illness. Inflammasome activation leads to release of IL-1β, IL-18, EVs and DAMPs, which results in barrier disruption and microbial translocation, as well as pyroptosis, systemic inflammation and immunothrombosis. These events trigger tissue- and organ-specific damage and dysfunction. Abbreviations: ARDS: Acute Respiratory Dystress Syndrome; BBB: Blood Brain Barrier; DAMPs: damage-associated molecular patterns; EVs: extracellular vesicles; IL-1β: interleukin-1 beta; IL-18: interleukin-18. Created with BioRender.com

NLRP3-driven inflammation contributes to organ dysfunction across multiple systems in critical illness (Fig. 2), although its manifestations vary depending on tissue-specific susceptibility, local immune environments, and the nature of the causative insult. Importantly, organ injury is rarely isolated; instead, inflammasome activation promotes inter-organ crosstalk, amplifying systemic dysfunction and contributing to the progression of MODS [92].

#### Lungs

Pulmonary injury represents the most extensively studied consequence of inflammasome activation in critical illness. In acute lung injury (ALI) and ARDS, excessive inflammasome activation has been consistently linked to disease severity and adverse outcomes. Elevated circulating and alveolar levels of IL-18 as well as other inflammasome biomarkers (e.g. caspase-1 and NLRP3) correlate with increased mortality, prolonged mechanical ventilation, and impaired oxygenation across diverse aetiologies [58, 59, 93–97]. Notably, local inflammasome activation within the pulmonary compartment appears to correlate more strongly with pulmonal disease severity than systemic markers [98]. In addition, increased IL-18 levels in bronchoalveolar lavage fluid have been associated with the development of secondary pulmonary infections, suggesting a link between dysregulated inflammasome signalling and impaired local host defence [99].

#### Kidneys

The role of the inflammasome in acute kidney injury (AKI) is supported primarily by preclinical studies, which consistently identify NLRP3 as a central mediator downstream of various initial triggers of AKI, including sepsis and rhabdomyolysis [100, 101]. Inflammasome activation in renal tubular epithelial cells with the typical caspase-1 activation and pyroptotic cell death, leads to tubular injury and loss of renal function [101–106]. Human biopsy data, although scarce, demonstrate co-localization of active caspase-1 and ASC with sites of tissue damage, supporting the clinical relevance of these findings [107].

#### Heart

Strong preclinical evidence indicates that inflammasome activation is a key player in cardiac dysfunction in critically ill patients via multiple mechanisms, including direct cytokine effects, pyroptosis, mitochondrial dysfunction and autophagy suppression [49, 108–115].

However, the available evidence on the pathophysiology in critically ill patients remains limited to date. A study conducted on patients suffering from sepsis revealed no correlation between IL1β or IL-18 and cardiac dysfunction, whereas traditional cardiac biomarkers exhibited a significant correlation. However, increased inflammasome markers were associated with mortality [116]. Other studies found a correlation between IL-1β and all-cause mortality after one year in patients with acutely decompensated heart failure [117]. In addition, circulating ASC specks in patients with cardiogenic shock have been demonstrated to serve as a prognostic marker for mortality [118].

#### Brain

Neuroinflammation is increasingly recognized as a contributor to acute brain dysfunction in critical illness, including sepsis-associated encephalopathy [119–123]. It may also lead to long-term cognitive impairment in survivors of critical illness [124].

Central mechanisms of central nervous system (CNS) damage in preclinical models are disruption of the blood-brain barrier mediated by endothelial cell pyroptosis and synaptic loss resulting from the release of IL-1β-enriched microvesicles. Both effects could be supressed through the inhibition of caspase-1 [121, 125, 126].

Evidence on inflammasome involvement in sepsis-associated encephalopathy is still inconsistent and thus inconclusive. Serum IL-1β has been found to be elevated in critically ill patients with delirium, irrespective of an underlying septic aetiology [127]. While a study of patients with sepsis revealed elevated levels of IL-1β in those who developed septic encephalopathy and a subsequent decrease in IL-1β levels upon resolution of encephalopathy [128], a larger multicentre study was unable to reproduce these results. However, the latter trial did find other proinflammatory cytokines to be associated with cognitive impairment [129]. A meta-analysis incorporating 40 studies that measured IL-1β in critically ill post-operative patients, once more demonstrated a correlation between delirium and IL-1β levels [130].

#### Gut

In preclinical models, inflammasome activation leads to intestinal mucosal barrier damage via pyroptosis following the upregulation of multiple inflammasome components through miR-155 as well as via increased tight junction permeability [131–136]. This facilitates the translocation of pathogens, toxins and PAMPs that foster infection spread, increase pathogen load and drive further inflammasome activation [137–139].

In critically ill patients elevated IL-1β levels are associated with intestinal barrier disruption [140]. This is in line with findings from chronic diseases, in which strong evidence indicates that the NLRP3 inflammasome contributes to intestinal dysfunction [141–143].

#### Liver

Patients with fulminant liver failure displayed elevated levels of IL-18 in both serum and hepatic tissue compared to milder liver dysfunctions, suggesting the involvement of the inflammasome in pathogenesis [144, 145]. Furthermore, elevated levels of IL-1β have been observed in patients suffering from acute liver failure, substantiating an involvement of the inflammasome in critical liver dysfunction [146–148].

From a mechanistic perspective, pyroptosis once again appears to be a significant catalyst in the process of inflammasome-induced organ. Findings from preclinical models and the assessment of gasdermin D in human hepatic tissue samples support this hypothesis [149, 150].

#### Organ cross-talk: inflammatory axes

Several inflammatory axes involved in numerous conditions have been described, linking different organs: the liver and the brain [139, 151–157], the lungs and the gut [151, 152], the heart and the kidneys [158], and the kidney, the heart and the lungs [159, 160]. While these organ-organ interactions are just beginning to be understood, their impact on multi-organ failure seems evident. Likewise, an involvement of inflammasome signalling in connection with microbial factors is emerging, i.e. a microbiota-inflammasome axis, as summarized in [161].

### Self-reinforcing cycle of inflammation

In patients with hyperinflammation, inflammasome activity is perpetuated and amplified by a self-reinforcing cycle that includes the aforementioned organ cross-talk. Although most of our understanding of this self-reinforcing cycle rests on evidence from preclinical models or chronic diseases, observations in critically ill patients support this notion [162–167]. The most common mechanism is the increase in circulating DAMPs from damaged organs that continues to activate the inflammasome and leads to further organ damage and, consequently, even more DAMP release. The amount of circulating PAMPs is increased by secondary infections caused through impaired immune barriers, such as bacterial translocation from the intestines or by insufficient infection control due to inflammasome-driven immune paralysis [7, 168, 169]. Intriguingly, DAMPs and PAMPs may also be transmitted by extracellular vesicles (EVs) [29], providing an alternative mode of signal dissemination. Recently, EVs have been reported to serve as vectors for transplantation of active GSDMD pores to neighbouring cells, providing a novel means for the direct transmission of pyroptosis from cell to cell [28].

Furthermore, cells that undergo pyroptosis release ASC specks into the extracellular space, where these add to the maturation of cytokines via caspase-1. Moreover, when internalized by macrophages, specks also lead to caspase-1 activity in these cells via a prion-like mechanism [170, 171].

Another amplification mechanism is based on mitochondrial dysfunction. As a consequence of the activation of caspase-1 or the occurrence of cellular stress, the mitochondrial membrane becomes permeable to molecules that serve as DAMPs (mtDAMPs). These activate the inflammasome directly, with mitochondrial DNA being of particular importance due to its ability to activate the inflammasome indirectly via the cGAS-STING pathway. Furthermore, the release of ROS from dysfunctional mitochondria also contributes to inflammasome activation [46, 47, 49, 72, 172–174].

Inflammasome activity is closely linked to the NF-κB signalling pathway. IL-1β and IL-18 can activate the NF-kB pathway and lead to transcriptional upregulation of inflammasome components and pro-interleukins. Interestingly, NF-kB also seems to have anti-inflammatory properties that inhibit NLRP3 activation via mitophagic clearance of damaged mitochondria. However, this effect might be limited, when mitochondrial damage is overwhelming [175, 176]. A further effect of IL-1β and IL-18 is the recruitment of additional immune cells that amplify inflammation via respiratory burst (release of ROS) or NETosis with the release of DAMPs [46, 177].

Finally, NLRP3 inflammasome activation induces metabolic reprogramming, with a Warburg effect-like shift of cellular energy generation from oxidative phosphorylation to glycolysis, associated with accumulation of lactate. Lactate either functions as direct inflammasome activator or mediates this effect via an intracellular pH shift, leading to mitochondrial dysfunction and ROS generation [178–183]. Furthermore, lactate itself exhibits cytokine-maturation activity, which provides IL-1β and IL-18 independently of caspases [179].

### Dynamics and heterogeneity of inflammasome activity

Physiological inflammasome activity exhibits a specific pattern. Firstly, the inflammasome is moderately activated to the extent necessary for effective immunological responses against pathogen invasion, or to facilitate healing processes in damaged tissues. This moderate activity is maintained until the resolution of the initial injury or infection. Once accomplished, the inflammasome activity rapidly diminishes and ultimately reverts to steady-state homeostasis, i.e. a state analogous to the original condition preceding inflammasome activation [7, 81, 162, 184]. Of note, inflammasome activity constitutes one of numerous interacting pro- and anti-inflammatory processes, which remain insufficiently understood [185–187].

In critically ill patients, inflammasome activity regularly deviates from the physiological pattern. The initial phase of the dysregulated pathway is often characterised by strong inflammasome activation in response to substantial tissue damage or a high infectious burden [56–60, 188], which may overshoot through amplification mechanisms detailed above. In addition, inflammasome hyperactivation can lead to immune exhaustion, resulting in subsequent immunosuppression [7, 35, 189]. Conversely, prematurely decreasing inflammasome activity before resolution of the underlying pathology leads to insufficient immune responses [35, 162] and prolonged inflammasome activation to persistent immunosuppression and negative outcomes [81, 190].

Previously immunosuppressed patients may exhibit insufficient inflammasome activation throughout their disease course. While this could theoretically result in increased mortality due to impaired pathogen clearance, current data on this specific patient subgroup are insufficient, representing an important knowledge gap [82].

**Fig. 3:**
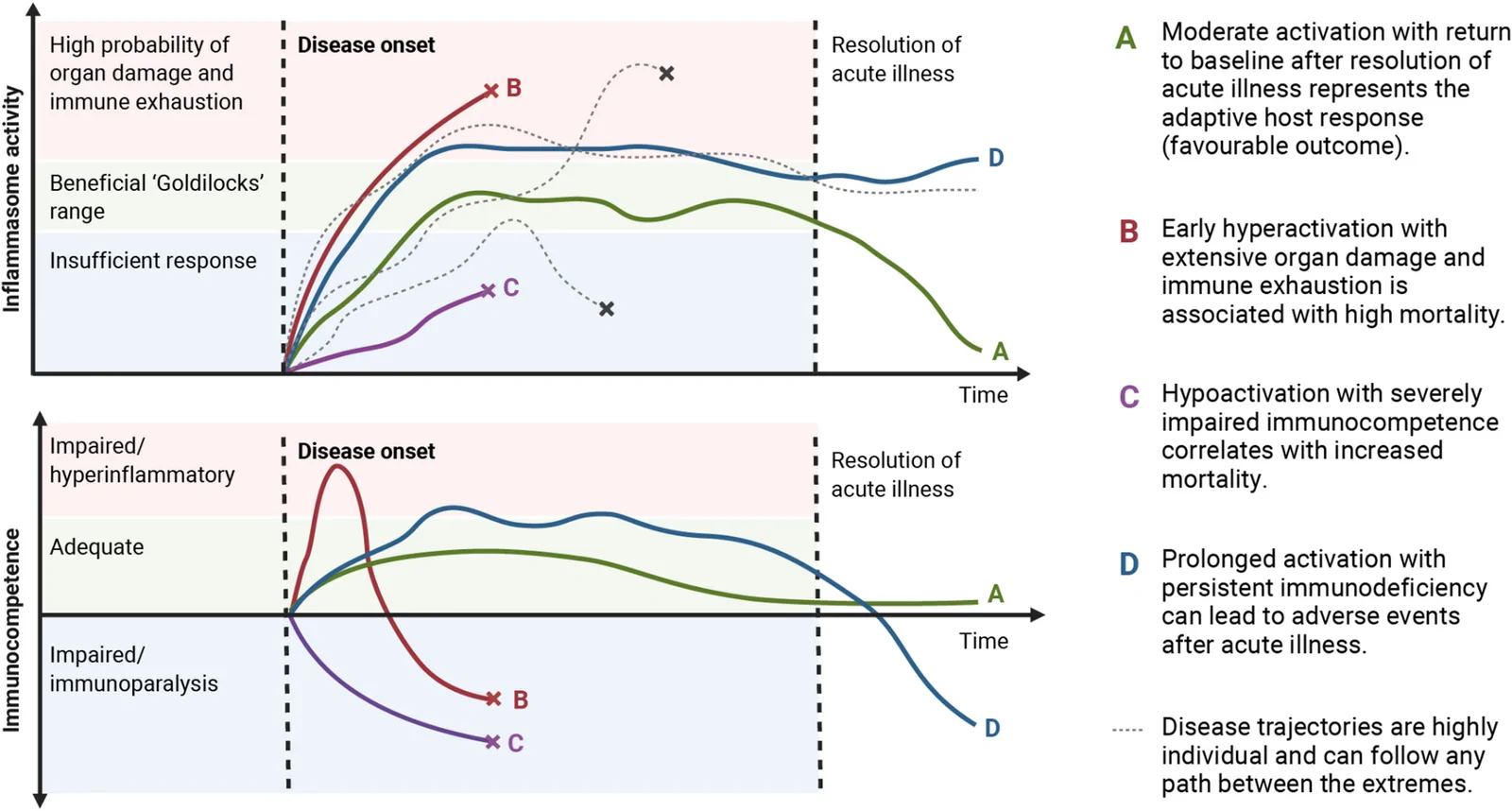
Temporal trajectories of host immune response following acute illness and associated outcomes. The upper panel depicts immune response trajectories over time relative to the onset and resolution of acute illness (vertical dashed lines), highlighting states of excessive inflammation, balanced (“Goldilocks”) immune activity, and immune suppression. The lower panel summarizes corresponding immune competence states ranging from hyperinflammation to immunoparalysis and their evolution after clinical recovery. Currently, the depicted “Goldilocks range” merely is a conceptual framework rather than a quantitatively defined therapeutic window, as validated biomarker thresholds for optimal inflammasome activity over time have not yet been established. Created with BioRender.com

In view of the deleterious consequences of both hypo- and hyperactivation of the inflammasome, any potential therapeutic approach must enable an optimal level of inflammasome activation, followed by a subsequent resolution phase. This aligns with a ‘Goldilocks principle’ (Fig. 3) of beneficial inflammasome activation [7, 10, 16, 81, 191]. At present, no validated biomarker panels or quantitative thresholds are available to define optimal inflammasome activity across the heterogeneous population of critically ill patients. This is indicative of the considerable temporal and interindividual heterogeneity of immunological states, including inflammasome activity. Consequently, defining a universal optimal range is challenging. The proposed ‘Goldilocks principle’ must be regarded as a conceptual framework at this time, which will require expansion by quantitative thresholds to enable clinical operationalization once the necessary evidence has been obtained.

Deviations from the beneficial path in either direction are highly individual and may change over time. Differences in genetic background, age, comorbidities, pathogen characteristics and the magnitude of tissue damage contribute to these heterogeneous trajectories. Genetic polymorphisms affecting inflammasome components and regulators, as well as variations in baseline immune competence and inflammatory status, may be especially important and have the capacity to induce both protective and detrimental effects [37, 55, 192–209].

The diverse temporal dynamics highlight a critical limitation of static biomarker assessment, as baseline measurements do not reflect changing or unstable immune states in critically ill patients. This issue can be addressed by serial measurements of inflammasome markers [16, 162, 210]. The considerable heterogeneity of individual trajectories is of particular clinical importance, as it likely aggravates the inconsistent observations in trials of immunomodulatory therapies.

It also emphasizes the often stated need for stratified treatment approaches based on an improved knowledge of the biology of the host response, thereby accounting for patient-specific immune profiles rather than applying uniform treatment strategies across heterogeneous populations [16, 82, 186, 187, 211].

### Clinical stratification based on biomarkers

The heterogeneous patterns of inflammasome activity are best reflected in the two prototypical groups of endotypes in critical illness that would benefit most from immunomodulatory therapies.

Hyperinflammatory endotypes are characterised by elevated levels of IL-1β and IL-18, increased activity of caspase-1, enhanced pyroptosis, and an elevated risk of early organ dysfunction. This pattern is commonly observed in early sepsis, hyperinflammatory ARDS, and severe viral infections, such as Coronavirus-19 disease (COVID-19) [188, 207, 212, 213]. In contrast, hypoinflammatory endotypes exhibit reduced cytokine production, impaired monocyte function, and defective antigen presentation, predisposing patients to persistent infections and secondary complications [16, 214–217]. These variants are typically observed in the later stages of sepsis or during prolonged intensive care unit stays.

Importantly, these endotype classifications are not static, but rather vary or even shift within an individual patient over the course of disease [7, 16, 162, 190]. Additionally, these prototypic endotypes form the extremes of a continuum of activation states, while there is no sharp transition, in which hyperinflammation abruptly switches to hypoinflammation or vice versa. Overlapping endotypes have also been observed, characterised by both, a robust proinflammatory response and concurrent immunosuppression [81]. Furthermore, the inflammasome merely constitutes a single component within the intricate immunological network. The integrated response of the host may thus deviate from the direct effects exerted by the inflammasome. These complex dynamics present a significant challenge to clinical assessment and therapeutic decision-making.

Recent endotyping frameworks, such as the consensus transcriptomic subtypes (CTSs) for sepsis [218], enable the integration of inflammasome-related signatures into broader disease endotypes, facilitating a more comprehensive and biologically informed patient stratification.

Biomarkers of inflammasome activation represent a promising approach for risk stratification and patient endotyping, but implementation in clinical practice remains a challenge. Table 1 summarizes the inflammasome-related biomarkers and their proposed clinical applications in sepsis. Circulating markers such as IL-1β, IL-18, GSDMD cleavage products, NLRP3 and ASC specks provide accessible indicators of systemic inflammasome activity [55, 94, 188, 219–221]. Among these, IL-18 has emerged as the most consistent prognostic biomarker in critical illness [58, 93–95]. Modern diagnostic techniques, such as the transcriptional analysis of inflammasome components, have the potential to deepen our understanding of a patient’s inflammasome status, especially when integrated into high throughput transcriptomic approaches [162, 163]. Other omics technologies, including metabolomics and cytomics, along with AI-driven data analysis, have advanced significantly in recent years and may facilitate therapeutic decisions in the future by providing additional underlying data. However, their clinical applicability is limited by complexity, labour intensity, and lack of standardization [16].

Overall, integration of biomarker data with clinical context and disease trajectories will be essential for advancing biomarker-guided precision medicine approaches targeting inflammasome pathways in critically ill patients [12, 16, 212, 216, 217]. One indirect strategy to avoid the complexity of the above-mentioned approaches is to use biomarkers, which indicate over-activation of tissue macrophages linking to excess production of IL-1β. Ferritin appears the most promising biomarker of IL-1β overactivation. Blood levels more than 4,420 ng/ml have specificity and negative predictive value exceeding 98% to indicate excess inflammasome activation and high levels of IL-18 [222].

**Table 1:** Biomarkers of inflammasome activation with potential clinical utility for diagnosis, patient stratification and monitoring in sepsis

Marker	Significance / potential	Potential assays
IL-1ß	Early surge correlates with disease severity	ELISA, FACS-based immunoassays, mass spectrometry
IL-18	Sustained elevation correlates with organ dysfunction and mortality	ELISA, FACS-based immunoassays
Caspase-1	Reflects tissue injury	qPCR, transcriptome, mass spectrometry, fluorescent activity assays
Caspase-4/5	Component of atypical inflammasome; Indicator of immune suppression; associated with worse outcomes	qPCR, transcriptome
GSDMD	Indicator of pyroptosis and tissue damage	Mass spectrometry
ASC specks	Circulating inflammasome complexes; associated with worse outcomes	FACS
EVs	Possible mechanism of pyroptosis transmission and spread; only pre-clinical evidence so far	Nanoparticle tracking analysis, FACS
DAMPs released in pyroptosis	Mechanism of inflammation spread; some DAMPs (e.g. progranulin) associated with worse outcomes	ELISA, mass spectrometry

Abbreviations: IL: interleukin; GSDMD: Gasdermin D; ASC specks: apoptosis-associated speck-like protein containing a caspase recruitment domain specks; EVs: Extracellular Vesicles; DAMPs: damage-associated molecular patterns; FACS: Fluorescence-Activated Cell Sorting; ELISA: Enzyme-linked Immunosorbent Assay; qPCR: Real Time Quantitative PCR

### Therapeutic strategies targeting inflammasome pathways

**Fig. 4:**
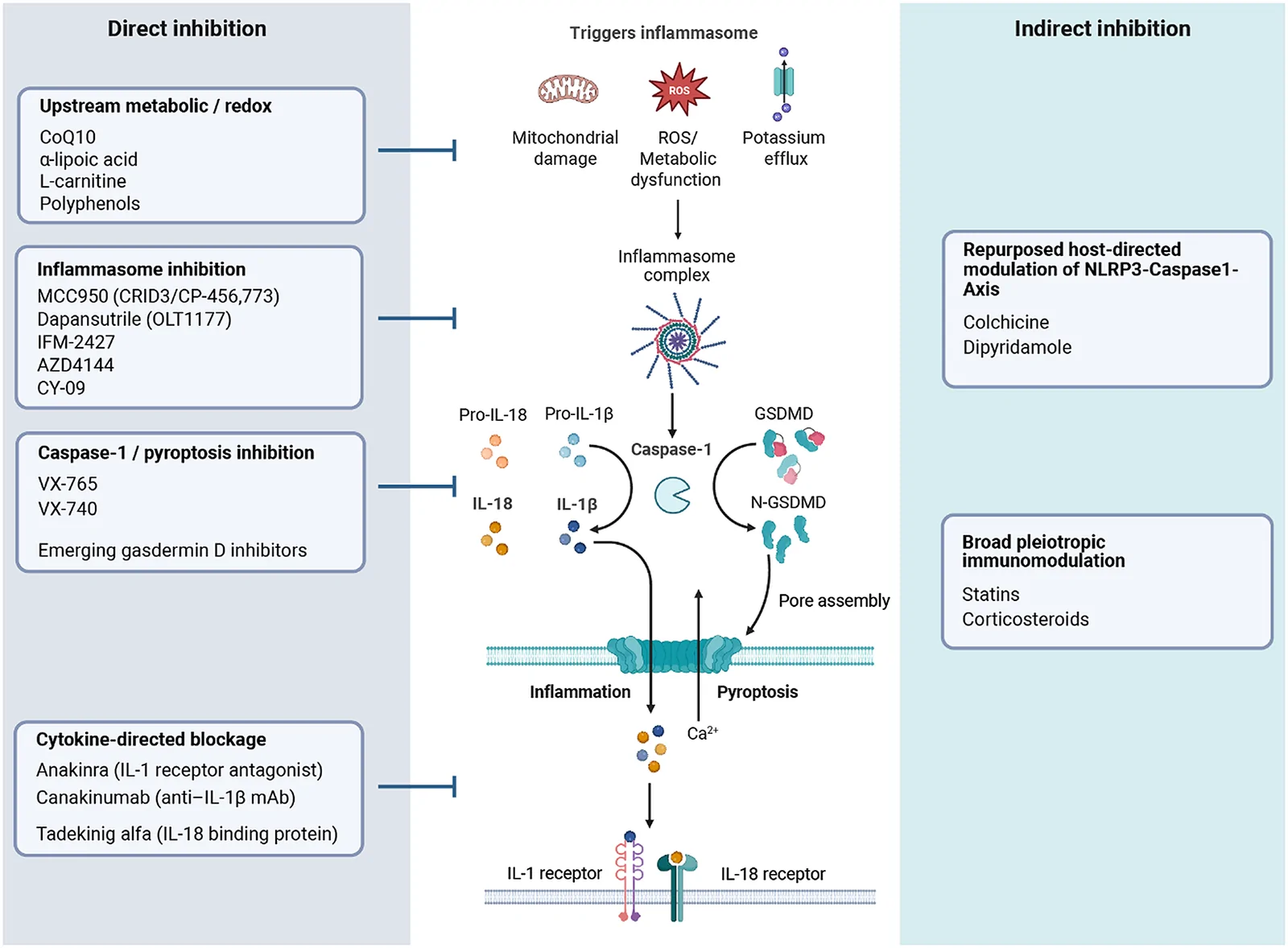
Inflammasome pathway–targeted treatments. Abbreviations: CoQ10: Coenzyme Q10; IL-1: interleukin-1; IL-1βmAb: interleukin-1 beta monoclonal antibody; NLRP3: NOD-, LRR- and pyrin domain–containing protein 3. Created with BioRender.com

Targeting inflammasome signalling offers a mechanistically plausible approach to modulate dysregulated inflammation in critical illness but requires careful balance between immunosuppression and preservation of host defence. These strategies can be categorized into six mechanistic levels and be distinguished by targets within the inflammasome pathway (Table 2; Fig. 4).

#### Direct inflammasome inhibition (NLRP3 inflammasome)

This represents the most upstream and direct strategy, aiming at blocking NLRP3 activation and assembly. Agents such as MCC950 (CRID3/CP-456,773), dapansutrile (OLT1177), IFM-2427, AZD4144, CY-09, and related triazinone derivatives directly suppress inflammasome activation, thereby reducing IL-1β and IL-18 release. MCC950 showed high specificity and efficacy for NLRP3 inhibition in murine models of inflammatory diseases [223]. However, clinical development was halted due to hepatotoxicity observed in animal models [224], while dapansutrile has reached phase II evaluation [225]. Overall, this class provides potent upstream inhibition but remains largely investigational.

#### Caspase-1 / pyroptosis inhibition (caspase-1; gasdermin D)

Intermediate strategies target inflammasome effector pathways, blocking cytokine maturation and pyroptotic cell death. VX-765 (VRT-043198 prodrug) and VX-740 (pralnacasan, discontinued due to toxicity) inhibit caspase-1, thereby reducing IL-1β and IL-18 activation [226]. Emerging GSDMD inhibitors directly prevent pore formation and pyroptosis, a key mechanism in inflammatory tissue injury and NET-driven organ dysfunction. To date, clinical translation remains limited by safety concerns and potential immunosuppression [227].

#### Cytokine-directed therapy (downstream blockade)

This clinically most advanced approach targets inflammasome downstream mediators. IL-1 signalling blockade with anakinra (IL-1 receptor antagonist) and canakinumab (anti–IL-1β monoclonal antibody) is supported by clinical evidence, including intensive care unit (ICU) use and randomized controlled trials (RCTs) data in selected hyperinflammatory phenotypes (e.g., MALS, severe COVID-19 subgroups) [12, 228]. A phase II study of tadekinig alfa in adult-onset Still’s disease shows overall good tolerability and initial evidence of efficacy through blockade of IL-18 [229], although its use remains experimental and requires biomarker-based patient selection due to potential risks, particularly in cases of low inflammatory activity.

#### Repurposed host-directed drugs (broad inflammasome modulators)

Repurposed agents, such as colchicine and dipyridamole, indirectly modulate the NLRP3/caspase-1 axis by attenuating inflammasome activation and downstream cytokine release. Strong clinical trial evidence exists on colchicine in cardiovascular disease [230–232], but data in infectious diseases and COVID-19 are limited [233]. These agents are widely available at low cost but provide only partial and non-specific inflammasome inhibition.

#### Upstream metabolic / redox modulation (inflammasome triggers)

This strategy targets upstream activators of inflammasome signalling, including ROS generation, mitochondrial dysfunction, and potassium efflux. Agents such as MitoQ, SkQ1, MitoTEMPO, CoQ10, α-lipoic acid, L-carnitine, and polyphenols reduce inflammasome priming signals. Evidence remains preclinical or based on mixed clinical trials, with variable efficacy depending on patient selection and disease context [234–236].

#### General immune modulators (pleiotropic drugs)

Broad anti-inflammatory therapies influence multiple pathways, including indirect modulation of inflammasome signalling. Among these, steroids exert complex and context-dependent effects mediated through both glucocorticoid and mineralocorticoid receptor pathways. Evidence supports the use of steroids in ARDS [237–239] and septic shock [240].

Glucocorticoids (e.g., dexamethasone, hydrocortisone) suppress inflammasome activity primarily at the priming stage by inhibiting NF-κB-dependent transcription of key components, such as NLRP3 and pro-IL-1β [241, 242]. In addition, they reduce the production of upstream inflammatory mediators, attenuate cytokine amplification loops, and may limit pyroptosis indirectly by decreasing caspase-1 activation. However, these effects are highly dependent on timing, dose, and the underlying immune phenotype, as excessive or prolonged glucocorticoid exposure may contribute to immunosuppression and impaired pathogen clearance [243].

Mineralocorticoids, particularly fludrocortisone, exert complementary effects through modulation of vascular tone, endothelial stability, and sodium-fluid homeostasis. Mineralocorticoid steroidogenesis was more frequently impaired in sepsis than glucocorticoid steroidogenesis in exploratory analyses [244]. Emerging evidence suggests that mineralocorticoid receptor signalling may also influence innate immune responses, including leukocyte activation and endothelial inflammation, thereby indirectly affecting inflammasome-driven pathways. In septic shock, combined glucocorticoid-mineralocorticoid therapy (e.g., hydrocortisone plus fludrocortisone) has been associated with improved haemodynamic stability and survival, potentially reflecting synergistic effects on both immune regulation and vascular responsiveness [240].

Statins (e.g., atorvastatin, simvastatin) represent another class of pleiotropic agents that may attenuate inflammasome activation through inhibition of cholesterol-dependent membrane signalling, reduction of oxidative stress, and suppression of NLRP3 assembly. So far, results on the benefit of statins in the treatment of sepsis remain ambiguous [245, 246]. However, statins were associated with decreased mortality in critically ill patients with sepsis in a recently published cohort study [247] and hyperinflammatory patients with ARDS in the HARP-2 trial [248].

Of note, these pleiotropic agents do not target the inflammasome directly but modulate its activity through upstream and systemic mechanisms. Their net effect is highly context-dependent, with potential to either restore immune homeostasis or exacerbate immunosuppression, underscoring the importance of endotype-guided application in critical illness.

Beside the pharmacological treatment options described, extracorporeal blood purification techniques remove not only PAMPs and DAMPs but also non-specific, medium-sized hydrophobic molecules, such as cytokines including IL-1β and IL-18. A RCT on hemofiltration during cardiac surgery of endocarditis patients [249] and a meta-analysis on patients with sepsis/septic shock [250] have failed to demonstrate a survival benefit associated with the use of these techniques. However, a large-scale RCT in septic shock patients was stopped because of possible harmful effects [251] and a trial on patients with severe COVID-19 pneumonia found increased mortality risk associated to early cytokine absorption [252].

**Table 2:** Inflammasome-targeting therapeutic strategies: clinical evidence from selected human observational and interventional studies across diseases

Mechanistic therapeutic axis	Molecular target	Core mechanism (signal-level summary)	Key agents	Clinical stage / evidence	Best suited clinical phenotype	Reference
1. Direct inflammasome inhibition	NLRP3 inflamma-some	Blocks inflammasome, resulting in ↓ caspase-1 activation ↓ IL-1β / IL-18 maturation	MCC950 (CRID3/CP-456,773), dapansutrile (OLT1177), IFM-2427, AZD4144, CY-09, triazinones	MCC950: preclinical (development limited by hepatotoxicity); dapansutrile: Phase II; others: early/preclinical	Early hyper-inflammation/ sterile systemic inflammation (pre-organ failure phase)	[225, 253]
2. Caspase-1 / pyroptosis inhibition	Caspase-1; gasdermin D	Blocks cytokine maturation and inhibits pyroptotic membrane pore formation and thus ↓ IL-1β/IL-18 release ↓ inflammatory cell death	VX-765 (VRT-043198), VX-740 (pralnacasan, discontinued), emerging gasdermin D inhibitors	VX-765: early clinical / advanced preclinical; VX-740: discontinued (toxicity); GSDMD inhibitors: preclinical	Sepsis with dominant pyroptosis/ tissue injury phenotype; organ dysfunction with high DAMP burden	
3. Cytokine-directed blockage	IL-1 signalling	Neutralization of effector cytokines interrupts systemic inflammatory amplification loop	Anakinra (IL-1 receptor antagonist) Canakinumab (anti–IL-1β mAb)	Anakinra: ICU/off-label and RCT-supported in subgroups Canakinumab: approved for autoinflammatory disease	Hyperinflamma-tory phenotypes (MALS sepsis, cytokine storm, severe COVID-19 inflammatory sub-phenotype)	[12, 228], 254– [256] [257–259]
4. Repurposed host-directed modulation	NLRP3 / caspase-1 axis (indirect)	Partial inhibition of inflammasome priming and amplification via cytoskeletal and platelet–immune interactions	Colchicine, dipyridamole	Colchicine: strong CV RCT evidence; mixed infectious/ COVID-19 data; others limited	Mild-to-moderate systemic inflammation; cardiovascular-risk overlap phenotypes; early infection phase	[230– [233, 260]
5. Metabolic / redox upstream modulation	Inflamma-some triggers (ROS, mitochondria, K⁺ efflux)	Reduce upstream danger signals and thus priming	Mitochondria-targeted antioxidants, CoQ10, α-lipoic acid, L-carnitine, polyphenols	Preclinical and heterogeneous early clinical data	Metabolic dysfunction-driven critical illness (mitochondrial dysfunction, shock states, post-ischemic inflammation)	[234–236]
6. Broad pleiotropic immunomodu-lation	Multiple inflamma-tory path-ways incl. NLRP3	System-wide immunomodulation produces transcriptional, endothelial and cytokine-level effects	Statins (atorvastatin, simvastatin), Corticosteroids (dexametha-sone, HC, FC)	Statins: mixed RCT/observational evidence Corticosteroids: strong ICU evidence (ARDS, septic shock)	Late hyperinflammation/ established ARDS/ septic shock with systemic inflammatory response	[245– [247, 261] [237–239] [240, 262–265]

Abbreviations: IL: interleukin; ROS: reactive oxygen species; MALS: macrophage activation-like syndrome; COVID-19: coronavirus disease-19, ICU: intensive care unit, CV: cardiovascular; RCT: randomized controlled trial; ARDS: acute respiratory distress syndrome; HC: hydrocortisone, FC: fludrocortisone; NLRP3: NOD-, LRR- and pyrin domain-containing protein 3; NETs: Neutrophil extracellular traps

### Clinical integration, individualized immunomodulation and outlook

Across all therapeutic strategies, efficacy strongly depends on the underlying immune phenotype and its temporal development during critical illness. Hyperinflammatory states are more likely to benefit from upstream inflammasome inhibition or downstream IL-1-targeted approaches, whereas patients with immunosuppression or immune exhaustion may be harmed by excessive pathway blockade. Thus, the clinical success of inflammasome-targeted therapies will depend on biomarker-guided stratification to achieving precise, context-dependent immunomodulation rather than global inhibition. A deeper understanding of temporal immune dynamics, disease-specific inflammasome activation patterns, and interindividual heterogeneity is essential. Future progress will rely on integrating biomarker-driven patient selection with longitudinal immune monitoring to guide timing and choice of therapy, ultimately enabling a precision medicine approach in critical care.

In this context, inflammasome biology provides a mechanistic framework for patient stratification and dynamic risk assessment. As an upstream regulator of IL-1β and IL-18 release, inflammasome signalling may identify patients early in the inflammatory cascade and capture transitions between hyperinflammatory and immunosuppressed states.

To accelerate clinical translation, several research priorities should be addressed. First, prospective longitudinal studies integrating serial measurements of inflammasome-related biomarkers (e.g., IL-18, caspase-1 activity, GDDMD fragments, and ASC specks) with clinical phenotyping and omics profiling are needed to define dynamic immune endotypes and identify optimal therapeutic windows for intervention. Second, biomarker-enriched adaptive clinical trials should evaluate inflammasome-targeted therapies in biologically selected patient populations rather than unselected cohorts, thereby increasing the likelihood of detecting clinically meaningful treatment effects. Third, the development and validation of rapid point-of-care assays capable of real-time assessment of inflammasome activity would facilitate bedside patient stratification and enable individualized immunomodulatory treatment. Together, these approaches have the potential to transform inflammasome biology from a mechanistic concept into a clinically actionable framework for precision medicine in critical illness (Table 3).

**Table 3:** Knowledge gaps and future directions in inflammasome-targeted treatment approaches

Domain	Current limitation	Needed advancements	Clinical implication
Biomarkers of inflammasome activation	Lack of standardization; low sensitivity of IL-1β measurement as a biomarker of inflammasome activation; variability across compartments	Development of validated biomarker panels (e.g., IL-18, caspase-1 activity, GSDMD fragments); point-of-care and real-time assays; integration with transcriptomics	Enables accurate patient stratification and monitoring of treatment response
Patient heterogeneity and endotypes	Poor definition of inflammasome-driven phenotypes; overlap of hyper- and hypoinflammatory states	Endotype classification integrating clinical data, biomarkers, and omics approaches; machine learning–based clustering	Identification of patients most likely to benefit from targeted therapies
Temporal dynamics of immune response	Static, single time-point measurements; unclear therapeutic windows	Longitudinal immune monitoring; dynamic biomarker-guided treatment algorithms	Optimizes timing of immunomodulatory interventions and reduces harm
Clinical trial design	Heterogeneous populations dilute treatment effects; negative RCTs despite biological rationale	Biomarker-enriched, adaptive/platform trial designs; stratified randomization based on immune phenotype	Improves signal detection and likelihood of successful translation
Therapeutic targeting specificity	Broad immunosuppression with pleiotropic agents; limited clinical data for selective inhibitors	Development of selective NLRP3, caspase-1, and GSDMD inhibitors with robust safety profiles; combination strategies	Enables precise modulation without impairing host defence
Safety and immunosuppression risk	Risk of secondary infections and delayed pathogen clearance	Risk stratification tools; integration of immune function assays (e.g., monocyte HLA-DR)	Balances anti-inflammatory effects with preservation of immunity
Organ-specific responses and compartmentalization	Limited understanding of tissue-specific inflammasome	Compartment-specific studies (e.g., lung vs. blood); imaging and tissue biomarkers	Supports organ-targeted therapies and improves outcome prediction
Translational integration	Gap between experimental findings and bedside application	Standardized protocols linking mechanistic biomarkers with clinical endpoints; PK/PD-guided approaches	Accelerates translation into routine ICU practice

Abbrev.: IL: interleukin, GSDMD: gasdermin D, RCT: randomized controlled trial, HLA-DR: human leukocyte antigen, PK: pharmacokinetics, PD: pharmacodynamics; ICU: intensive care unit; NLRP3: NOD-, LRR- and pyrin domain-containing protein 3

## Conclusions

The inflammasome emerges as a central regulator of the host response, essential for effective innate immunity but capable of driving pathological inflammation and immune dysfunction when dysregulated, thereby contributing fundamentally to the pathobiology of critical illness. Future therapeutic success will depend on precision strategies that align patient selection, biological phenotype, and timing of intervention to restore immune balance rather than indiscriminately suppress inflammation.

## Data Availability

No datasets were generated or analysed during the current study.

## Abbreviations

AIM2: Absent in melanoma 2
AKI: Acute kidney injury
ALI: Acute lung injury
ARDS: Acute respiratory distress syndrome
ASC: Apoptosis-associated speck-like protein
ATP: Adenosintriphosphate
CAPS: Cryopyrin-associated periodic syndromes
CNS: Central nervous system
COVID-19: Coronavirus-19- disease
CASP: Caspase
CT: Consensus transcriptomic subtypes
DAMPs: Damage-associated molecular patterns
ELISA: Enzyme-linked Immunosorbent Assay
EVs: Extracellular vesicles
FACS: Fluorescence-Activated Cell Sorting
FC: Fludrocortisone
GSDMD: Gasdermin D
HC: Hydrocortisone
HMGB1: High Mobility Group Box 1
HLA-DR: Human Leukocyte Antigen – D Related
ICU: Intensive care unit
IFNB: Interferon beta
ILs: Interleukins
IL1R: Interleukin-1 receptor
LPS: Lipopolysaccharide
MALS: Macrophage activation-like syndrome
MAVS: Mitochondrial antiviral-signalling protein
MODS: Multiple organ dysfunction syndrome
NET: Neutrophil extracellular traps
NF-κB: Nuclear Factor kappa-light-chain-enhancer of activated B cells
NLRP3: NOD-, LRR- and pyrin domain-containing protein 3
NLRC4: NLR family CARD domain-containing protein 4
oxmtDNA: Oxidized mitochondrial DNA
PAMPs: Pathogen-associated molecular patterns
PD: Pharmakodynamics
PK: Pharmakokinetics
P2X7: Purinergic receptor P2X ligand-gated ion channel 7
qPCR: Real Time Quantitative PCR
RCD: Regulated cell death
RCT: Randomized controlled trial
PRR: Pattern recognition receptors
ROS: Reactive oxygen species
TLR: Toll-like receptors
TNF: Tumor necrosis factor
TNFR: Tumor necrosis factor receptor
TWIK2: Tandem of P domains in a weak inwardly rectifying K⁺ channel 2
